# Les tumeurs malignes anorectales en milieu hospitalier à Ouagadougou: aspects épidémiologiques et diagnostiques

**DOI:** 10.11604/pamj.2014.18.26.3003

**Published:** 2014-05-07

**Authors:** Alice Nanelin Guingané, Roger Arsène Sombié, Alain Bougouma

**Affiliations:** 1 Centre Hospitalier Universitaire Yalgado Ouédraogo (CHUY-O), Ouagadougou, Burkina Faso; 2 Unité de Formation et de Recherche en Sciences de la Santé (UFR-SDS), Université de Ouagadougou, Ouagadougou, Burkina Faso

**Keywords:** Tumeurs malignes, pathologie anorectale, épidémiologie, diagnostic, Burkina Faso, malignancies, anorectal disease, epidemiology, diagnosis, Burkina Faso

## Abstract

Le but de notre étude était de décrire les caractéristiques épidémiologiques et diagnostiques des tumeurs malignes anorectales en milieu hospitalier à Ouagadougou. Il s'est agi d'une étude rétrospective et transversale qui a concerné les patients vus en endoscopie digestive basse au cours de la période allant du 29/09/1999 au 04/10/2008. À l'aide d'une fiche de collecte, nous avons recueilli, dans 4 structures sanitaires et 3 laboratoires d'anatomie et de cytologie pathologiques de la ville de Ouagadougou, les données à partir des comptes-rendus d'endoscopie digestive basse et des registres d'anatomie et de cytologie pathologiques. Durant la période de notre étude, 645 patients ont été examinés en anorectoscopie et 882 cas d'affections anorectales colligés. Les tumeurs malignes anorectales avec 61 cas (6,9%) occupaient la quatrième place après la maladie hémorroïdaire (45,6%), les anites (21,1%) et les fissures (13,9%). Elles regroupaient les cancers du rectum (4,2%) et les cancers de l'anus (2,7%). Vingt cancers anorectaux ont été histologiquement confirmés parmi lesquels l'adénocarcinome était le type histologique le plus retrouvé avec 17 cas. Les tumeurs malignes, quatrième affection anorectale la plus fréquente au cours de notre étude, constituent une préoccupation du fait de leur fréquence croissante, leur diagnostic souvent tardif et les difficultés liées à leur prise en charge surtout dans nos pays avec une population à faible revenu. La sensibilisation de la population et la prescription plus large de l'endoscopie digestive basse devraient permettre une meilleure prise en charge des patients.

## Introduction

Les tumeurs malignes anorectales sont des affections dysmitotiques de l'anus et du rectum. Les cancers du canal anal sont rares, représentant et 3 à 4% des cancers colorectaux [1]. Leur présentation polymorphe, parfois faussement rassurante, retarde trop souvent le diagnostic malgré une situation anatomique immédiatement accessible. Pathologie fréquente et lourde, les cancers du rectum entrent dans le cadre des préoccupations de santé publique avec une fréquence globale élevée dans les pays d'Europe Occidentale, d'Amérique du nord et d'Océanie où le risque est voisin de 5% dans la population générale. L'incidence la plus faible est retrouvée en Asie, en Amérique du Sud, et en Afrique (moins de 10 pour 100 000 habitants). En Afrique, dans des études parcellaires, la région sub-saharienne apparaît comme celle où on rencontre les taux les plus faibles au monde [2, 3]. L'endoscopie digestive basse est incontournable dans le diagnostic du cancer anorectal, en ce sens qu'elle permet la visualisation de la lésion et la réalisation des biopsies pour le diagnostic histologique.

## Méthodes

Il s'est agi d'une étude rétrospective et transversale, couvrant la période du 29/09/1999 au 04/10/2008 soit 9 ans 5 jours. Elle a été réalisée dans les unités d'endoscopie du Centre Hospitalier Universitaire Yalgado Ouédraogo (CHU-YO) de Ouagadougou, de 2 cliniques privées et d'un centre confessionnel. Les malades qui y ont été reçus venaient de la ville de Ouagadougou et des provinces environnantes. Ont été inclus dans l’étude, les patients de tous âges, des deux sexes, de toutes nationalités et de toutes catégories socioprofessionnelles chez qui était suspecté une pathologie anorectale.

L'endoscopie digestive basse a été réalisée par 3 gastro-entérologues. Le matériel était composé de vidéocoloscopes OLYMPUS Evis Exera CF Q145 L, OLYMPUS Evis Exera CF Q160 AL, OLYMPUS CF 100 HI, PENTAX EC3840, FUJINON EC201WL; d'anuscopes pédiatriques et adultes à usage unique stériles, métalliques réutilisables adultes A. LEGRAND alimentés par une source lumineuse OLYMPUS Evis Exera CLV-160, de rectoscopes rigides, de sigmoïdoscopes HEINE OPTOTECNIK alimentés par une source lumineuse Heine HK 7000. La désinfection du matériel réutilisable s'est faite dans quatre bacs contenant respectivement un décontaminant (Cytéal^®^), de l'eau propre, du glutaraldéhyde (Endosporine^®^, Stéranios^®^ 2%) puis de l'eau propre.

## Résultats

Durant la période de notre étude, 645 patients ont été examinés en anorectoscopie et 882 cas d'affections anorectales colligés. Les tumeurs malignes anorectales avec 61 cas (6,9%) étaient la 4ième pathologie anorectale la plus fréquente (Tableau 1). Elles regroupaient les cancers du rectum (4,2%) et les cancers de l'anus (2,7%). Il s'agissait en majorité de femmes (56,76%) avec un sex-ratio de 1,32 pour les patients présentant un aspect dysmitotique rectal à l'endoscopie; par contre, il y'avait deux fois plus d'hommes (16) que de femmes, soit un sex-ratio de 2 pour ceux présentant une atteinte anale. L’âge moyen des patients était de 34,69 ans avec des extrêmes de 1 et 80 ans. Les cadres moyens (28,6%), les femmes au foyer (19%) et les élèves et étudiants (16,7%) étaient les plus représentés. Le tableau clinique présenté par les patients était dominé par l'hémorragie digestive basse (35,2%), la masse rectale (20,4%) et la proctalgie (12,5%). Les masses bourgeonnantes ont été l'aspect endoscopique le plus retrouvé avec 41 cas (67,2%); puis les masses ulcéro-bourgeonnantes et ulcérées avec respectivement 17 cas (27,9%) et 3 cas (4,9%). La localisation latérale gauche avec 12 cas (40%), était deux fois plus fréquente que chacune des autres localisations: latérale droite, antérieure ou postérieure. Des prélèvements chez 23 patients ont bénéficié d'une étude histologique et 20 cancers anorectaux ont été histologiquement confirmés parmi lesquels l'adénocarcinome était le type histologique le plus retrouvé avec 17 cas (Tableau 2). La concordance histo-endoscopique était de 87%.

**Tableau 1:** Les affections anorectales colligées chez 645 patients vus en anorectoscopie

Affections anorectales	Fréquence (n)	Pourcentage(%)
Les hémorroïdes	402	45,6
Les anites	186	21,1
Les fissures	123	13,9
Les tumeurs malignes anorectales	61	6,9
Les suppurations ano-périnéales	50	5,6
Les polypes de l'anus	25	2,8
Les rectites	19	2,2
Les polypes du rectum	10	1,1
Les prolapsus rectaux	6	0,7
**Total**	**882**	**100**

**Tableau 2:** Les types histologiques des cancers anorectaux diagnostiqués au cours de l’étude

Type histologique	Fréquence (n)	Pourcentage (%)
Adénocarcinome bien différencié liberkühnien du rectum	7	35
Adénocarcinome moyennement différencié liberkühnien rectum	5	25
Carcinome en bague à chaton du rectum	3	15
Adénocarcinome liberkühnien infiltrant du rectum	1	5
Adénocarcinome liberkühnien du rectum avec des emboles vasculaires	1	5
Adénocarcinome bien différencié infiltrant du rectum plus métastases ganglionnaires	1	5
Adénocarcinome mucineux du rectum	1	5
Adénome tubulaire avec des atypies cellulaires modérées du rectum	1	5
Total	20	100

## Discussion

**Figure 1:**
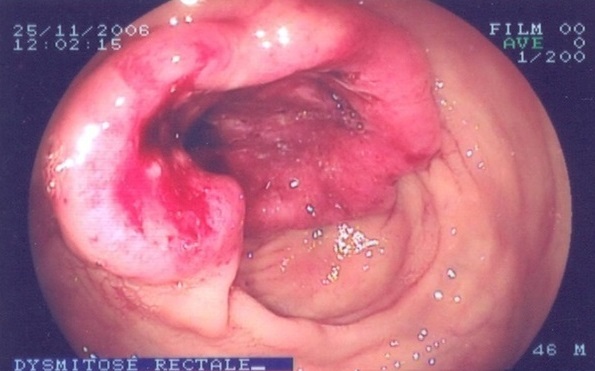
Dysmitose rectale vue en endoscopie chez un homme de 46 ans

**Figure 2:**
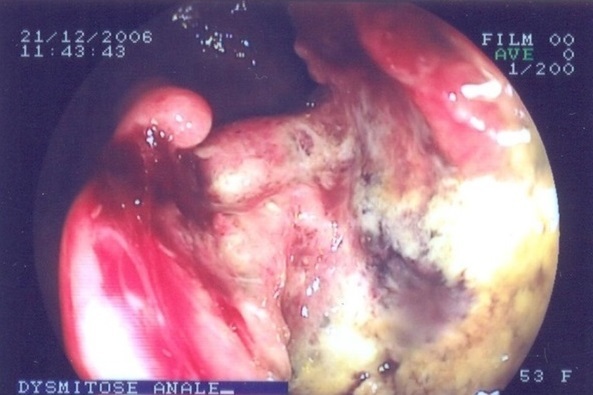
Dysmitose anale vue en endoscopie chez une femme de 53 ans

Rappelons que durant la période de notre étude, 61 cas de tumeurs malignes anorectales ont été suspectés à l'endoscopie soit 6,9% des affections anorectales et 20 cancers ont été histologiquement confirmés dont 17 au rectum (Figure 1) et 3 à l'anus (Figure 2). D'autres auteurs en Afrique ont, selon le recrutement, l'aire géographique et la période, rapporté des fréquences variables pour le cancer du rectum: Edino [4] au Nigéria, 50 cas en 4 ans. Padonou [5] au Bénin, 4 cas en 7ans et sani [6] au Niger, 30 cas en 12 ans. Pour le cancer de l'anus: Yassibanda [3], a retrouvé en 5 ans, 13 cas. Le cancer anorectal semble rare au Burkina et cette rareté est aussi observée en Afrique et dans le monde [1, 2, 5, 7–20]. L’âge moyen des patients était de 35 ans (15 et 72 ans). Gassaye [10] à Brazaville, retrouvait un âge moyen: 49 ans (40 et 59 ans). Amegbor [2] au Togo, a observé un âge moyen de 49 ans. Le jeune âge des patients a été trouvé par de nombreux auteurs en Afrique et il variait entre 40 et 53 ans [5, 12, 18, 21]; il serait lié entre autres: à la faible espérance de vie en Afrique et la jeunesse de la population, la présence d'importants facteurs de risque en raison de la pauvreté. Pour les cancers confirmés du rectum, nous avons noté une prédominance du sexe féminin avec 11 femmes (64,7%) pour 6 hommes (35,3%). Le sex-ratio était de 1,83. Sani [6] au Niger retrouvaient par contre une prédominance masculine tout comme la plupart des auteurs[4–6]. Cependant, nous pensons comme beaucoup d'auteurs que le cancer du rectum est indépendant du sexe. Concernant le cancer anal, nous retrouvions deux hommes pour une femme, le sex-ratio était donc de 2. Cette prédominance masculine était également observée dans les études de Gassaye [10], Sani [6]. Dans la littérature, le cancer de l'anus survient généralement chez la femme [19, 22, 23]. Les cadres moyens (28,6%), les femmes au foyer (19%) et les élèves et étudiants (16,7%) étaient les plus représentés. Au sujet du cancer rectal, le bas niveau socio-économique a été noté par de nombreux auteurs car, c'est dans ces milieux que le dénuement financier et l'absence d’éducation à la santé, ne permettent pas la consommation de fruits et légumes qui ont un effet protecteur contre cette affection et favorisent la consommation d'aliments conservés par salaison ou fumaison. Toutefois en Europe, on observe une prédominance au niveau des classes aisées; ceci pourrait s'expliquer par une plus forte consommation de graisse animale dans cette population. Le niveau socio-économique ne semble pas avoir d'influence sur la genèse du cancer de l'anus dans la littérature. L'absence de données sur les antécédents de la majeure partie de nos patients ne nous permet pas de tirer une quelconque conclusion. Toutefois, les affections parasitaires à l'origine de rectocolites inflammatoires chroniques, la faible consommation de fruits et légumes, la consommation de graisses animales, les polypes adénomateux, la polypose adénomateuse familiale, le syndrome HNPCC ou syndrome de Lynch, le tabac, l'alcool, le défaut d'activité physique, les maladies inflammatoires chroniques de l'intestin constituent les facteurs prédisposants au cancer du rectum [10, 24–30]. Les condylomes, l'homosexualité masculine, le tabac, l'infection par le *Papilloma virus* et par le VIH sont les facteurs prédisposants au cancer de l'anus[25, 24, 31, 30, 29].

L'hémorragie digestive basse 16 cas (38,8%), la proctalgie 8 cas (25%) et la masse rectale 7 cas (21,88%) étaient les indications les plus fréquentes chez les patients. La prédominance des rectorragies a été observée par de nombreux auteurs [2, 5, 7, 12, 17, 32, 33]. Elles font du cancer du rectum, le seul cancer digestif ayant une symptomatologie qui permet un diagnostic précoce. Leur caractère récidivant est source d'angoisse et de stimulation à la consultation. Ainsi donc, devant toute rectorragie, des examens clinique et endoscopique s'imposent. En Afrique, toute symptomatologie proctologique et particulièrement la rectorragie, est abusivement rattachée à la maladie hémorroïdaire. Ce qui engendre des tradithérapies, facteurs de retard au diagnostic et de découverte de formes évoluées.

L'aspect endoscopique le plus fréquemment retrouvé était les tumeurs bourgeonnantes avec 12 cas (60%), en concordance avec les données de la littérature. Toutes les formes peuvent être rencontrées, fonction du type anatomique, de l'intervalle séparant le début des signes du diagnostic. Toutes les parties du rectum peuvent être le siège donc de lésions tumorales par contre le cancer de la marge anale n'est pas pris en compte dans les études de nombreux auteurs car il est aujourd'hui considéré comme faisant partie des tumeurs dermatologiques. L'adénocarcinome était le type histologique le plus rencontré avec 15 cas (94,4%). La prédominance de l'adénocarcinome au niveau rectal a été constatée par la grande majorité des auteurs [4–7, 17, 32, 34]; elle est due à la richesse de la muqueuse rectale en glande de Lieberkühn. Le carcinome, au niveau anal, était le type histologique le plus présent dans notre étude, comme pour la plus part des auteurs de par le monde [8, 16, 20, 35, 36]. Soulignons que la petitesse de notre série, pour le cancer anal, ne nous permet pas de tirer une conclusion. Parmi les 61 cas de tumeurs malignes anorectales suspectés à l'endoscopie, des biopsies ont été réalisées chez 37 patients, 23 prélèvements ont bénéficié d'une étude histologique et 20 cancers anorectaux ont été histologiquement confirmés. La concordance histo-endoscopique était de 87% ce qui nous permet d'affirmer que l'endoscopie joue un rôle majeur dans le diagnostic des cancers anorectaux; en ce sens qu'elle permet la visualisation de la lésion et la réalisation de biopsies pour le diagnostic histologique.

## Conclusion

Les tumeurs malignes, quatrième affection anorectale la plus fréquente au cours de notre étude, constituent une préoccupation du fait de leur diagnostic trop souvent tardif dans notre contexte en raison entre autres, de l'idée communément admise que toutes les affections anorectales se résument à la maladie hémorroïdaire qui ne saurait être traitée que par les tradithérapeutes. L'anorectoscopie est un moyen simple et peu onéreux de faire le diagnostic de ces tumeurs dont le pronostic est lié à la précocité du diagnostic.
